# Acceptability of the Pregnancy, Exercise, and Nutrition Research Study With Smartphone App Support (PEARS) and the Use of Mobile Health in a Mixed Lifestyle Intervention by Pregnant Obese and Overweight Women: Secondary Analysis of a Randomized Controlled Trial

**DOI:** 10.2196/17189

**Published:** 2021-05-12

**Authors:** Ellen M Greene, Eileen C O'Brien, Maria A Kennelly, Orna A O'Brien, Karen L Lindsay, Fionnuala M McAuliffe

**Affiliations:** 1 UCD Perinatal Research Centre School of Medicine University College Dublin, National Maternity Hospital Dublin Ireland; 2 Department of Pediatrics University of California Irvine, CA United States

**Keywords:** pregnancy, mHealth, nutrition, lifestyle, acceptability, app, mobile phone

## Abstract

**Background:**

Dietary interventions can improve pregnancy outcomes among women with increased BMI. Although the interest in mobile health interventions is growing, little is known about the acceptability of smartphone apps to support lifestyle interventions in such a cohort.

**Objective:**

We aimed to assess the acceptability of the pregnancy, exercise, and nutrition research study with smartphone app support (PEARS) and the use of mobile health in a mixed lifestyle intervention delivered to overweight and obese pregnant women.

**Methods:**

PEARS was a randomized controlled trial of a low glycemic index dietary intervention with exercise prescription and a smartphone app, which was delivered to pregnant women who were overweight or obese. Acceptability questionnaires were completed by the intervention group at 28 weeks of gestation (n=149) and at postintervention (n=123). Maternal characteristics were recorded (ie, age, ethnicity, BMI, socioeconomic status). Associations between maternal characteristics and acceptability of the intervention and app were analyzed using two-tailed *t* tests, Mann-Whitney *U* tests, chi-square test, and logistic regression. One-on-one semistructured interviews were conducted with a subcohort of the intervention participants (n=28) at 34 weeks of gestation, in which the participants shared their experiences of the PEARS intervention.

**Results:**

The intervention was generally accepted, with respondents agreeing that the diet was easy to follow (98/148, 68.5%), enjoyable (106/148, 74.1%), and affordable (110/148, 76.9%). Qualitative and quantitative results were consistent with each another, both demonstrating that app acceptability was high. The participants agreed that the app was enjoyable (96/120, 80.0%) and easy to use (116/119, 97.5%). Compared to those with tertiary education, those with lower education levels were more likely to enjoy the dietary changes (*P*=.04). Enjoyment of the app was associated with disadvantaged neighborhood deprivation index (*P*=.01) and higher BMI (*P*=.03).

**Conclusions:**

The PEARS intervention and use of a supportive smartphone app were accepted by pregnant women, particularly by those from vulnerable subgroups of this population.

**Trial Registration:**

International Standard Randomized Controlled Trial Number (ISRCTN) 29316280; https://www.isrctn.com/ISRCTN29316280

## Introduction

### Mobile Health as a Support in Lifestyle Interventions

Mobile health (mHealth) is the use of mobile technology such as smartphones, personal digital assistants, or other wireless devices as a tool for medical or public health care purposes [1]. mHealth technology is now growing in popularity as a component of research interventions for both pregnant and nonpregnant cohorts. mHealth as part of mixed lifestyle interventions has been successful in promoting weight loss and increasing physical activity levels in nonpregnant normal weight, overweight, and obese individuals [2,3]. The use of mobile devices in such interventions has been deemed acceptable and found to be highly compliant, particularly among overweight individuals [3]. Furthermore, mHealth-supported diet and exercise interventions in pregnancy have been deemed effective and acceptable among overweight and obese cohorts [4-8]. However, the majority of studies used mHealth in the form of text messaging, websites, and fitness trackers [4-8]. There is a paucity of data on the acceptability of the use of mHealth in the form of a smartphone app specifically to support a mixed lifestyle intervention in pregnancy.

### Use of mHealth Among Hard-to-Reach Populations

Through the use of mHealth, there is increased potential to provide support to those subgroups of the population who experience barriers in obtaining health care information, such as low education level and low income or residing in difficult-to-reach geographical locations. Lower socioeconomic groups have relatively higher levels of ill health, fewer resources, and are more likely to report having chronic health conditions such as diabetes [9,10]. If designed accordingly, mobile technology could be used to present supplementary dietary and physical activity information using easy-to-understand language, which is supported by graphics. A smartphone app is a convenient and economical tool for communication as well as a potential additional support for those with few resources to obtain health care information. However, a review of the use of pregnancy apps highlighted that women with low incomes were among the groups with the lowest rate of pregnancy app uptake [11]. In order to ensure that smartphone apps communicating diet and lifestyle information are reached to lower socioeconomic groups, lower-income populations should be considered in the app development and the acceptability of reliable pregnancy apps should be examined in such groups.

### Pregnancy, Exercise, and Nutrition Research Study With Smartphone App Support

The pregnancy, exercise, and nutrition research study with smartphone app support (PEARS) used mHealth in the form of a smartphone app to support a diet and exercise intervention aimed to reduce the incidence of gestational diabetes mellitus (GDM) [12]. GDM is a metabolic condition of glucose intolerance in pregnancy with negative implications for both the mother and the fetus, for which a high prepregnancy BMI is a risk factor [13-15]. Currently, 53% of the female Irish population are overweight or obese [10], highlighting the need for interventions targeting at-risk groups to reduce the incidence of GDM. Dietary and lifestyle approaches are effective in improving outcomes [16-18], and several diet and exercise interventions carried out in pregnant cohorts, thus far, have been deemed acceptable by participants [19,20]. However, only few interventions aiming to reduce the incidence of GDM in at-risk groups have incorporated a smartphone app into the intervention design and therefore, the acceptability of a smartphone app used in this context is unknown. As pregnancy app uptake differs across various population groups [11], maternal demographics should be considered when examining smartphone app acceptability.

### Aims and Objectives of This Study

We aimed to quantitatively and qualitatively examine participant acceptability and demographics associated with the acceptability of the (1) PEARS intervention and (2) PEARS diet and exercise smartphone app.

## Methods

### Study Design

This study was a secondary data analysis of pregnant women originally recruited as part of the PEARS between 2013 and 2016 at the National Maternity Hospital, Dublin, Ireland. The detailed methodology and results of the PEARS study have been previously published [12,21]. In brief, PEARS was a randomized controlled trial that evaluated the effect of a “healthy lifestyle package” on the incidence of GDM in 565 pregnant women who were overweight or obese. The intervention group (n=278) received low glycemic index (GI) dietary advice, a daily exercise prescription, and a study-specific smartphone app as well as standard obstetric care throughout pregnancy. The control group received standard obstetric care only. The primary outcome was the incidence of GDM among participants at 28 weeks of gestation for which no difference was observed between the groups [12]. Differences were noted between intervention and control groups in gestational weight gain and in the delivery rates of large-for-gestational-age infants [12].

### Ethical Approval

Institutional ethical approval for the PEARS study was granted by The National Maternity Hospital Ethics committee in October 2012.

### Patient Selection

Pregnant women were recruited from the National Maternity Hospital. The predefined, published protocol was followed [21]. Eligibility criteria included possession of a smartphone, age of 18-45 years, between 10 and 16 weeks of gestation, early pregnancy BMI≥25 kg/m² and ≤39.9 kg/m², singleton pregnancy, and an adequate level of understanding of the English language to give informed consent.

### Assessment of Maternal Characteristics

Maternal weight and height were measured at enrolment and BMI was calculated (kg/m²) and the women were categorized as overweight (25.0-29.9 kg/m²) or obese (≥30 kg/m²). Age, parity, and ethnicity were collected from medical charts. Education level was self-reported by participants and they were classified as having achieved tertiary education or not. Neighborhood deprivation was assessed according to the Pobal Haase-Pratschke (Pobal HP) Deprivation Index [22] using participants’ home addresses and was categorized as advantaged (score>0) or disadvantaged (score<0).

### The PEARS Smartphone App

#### App Development

**Figure 1:**
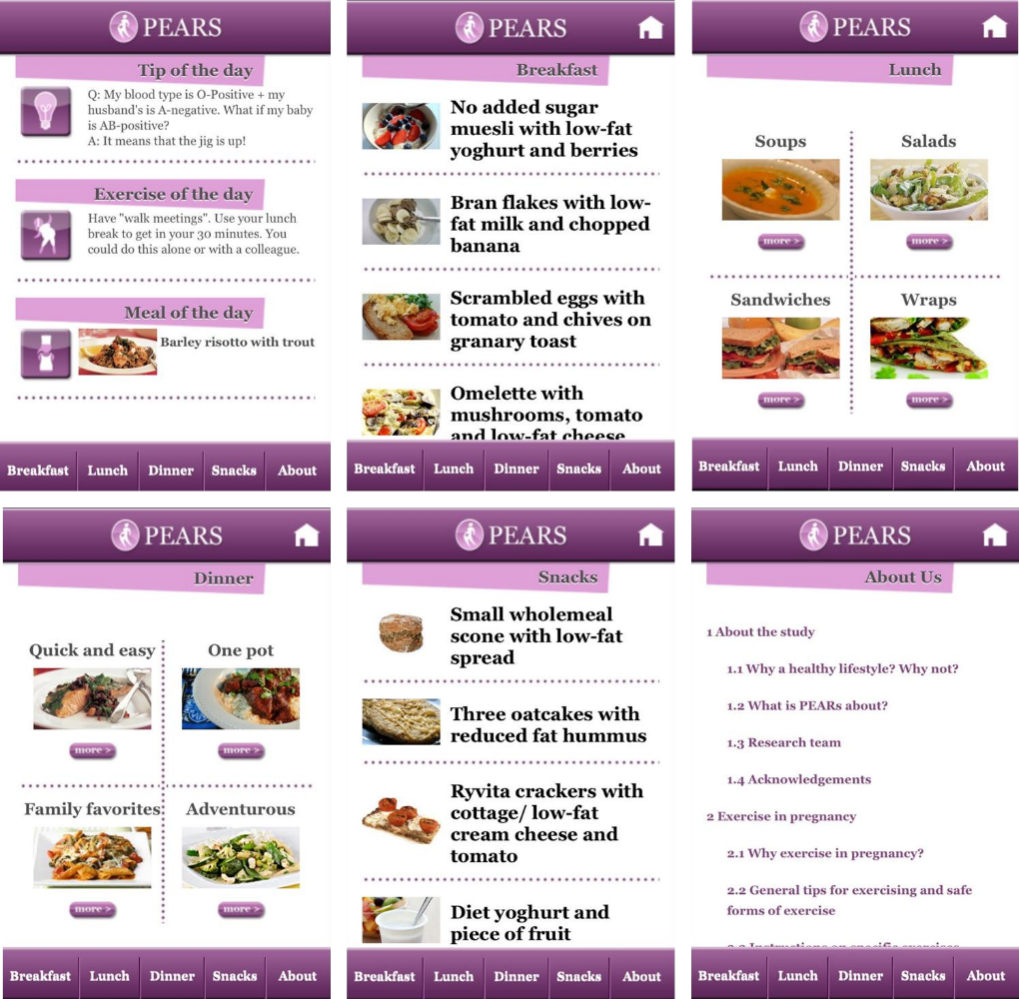
Screenshots of the pregnancy, exercise, and nutrition research study with smartphone app support (PEARS) app.

The smartphone app provided to the intervention group was a study-specific app designed to support the diet and physical activity information and advice provided by clinical research personnel during an in-person education session after enrolment (Figure 1). The smartphone app was designed by a multidisciplinary team, including a dietician, obstetrician, food behavior specialist, and an app design company. Focus groups were carried out among pregnant women during the development of the app to inform the app’s content. The focus groups were conducted by a research obstetrician and included 10 randomly selected pregnant women with an overweight or obese BMI. Information on these women’s knowledge of diet and exercise as well as their needs and wants in a lifestyle pregnancy app were used to develop the app’s design and content. The app’s text was developed for a reading age of 12 years to allow those with lower literacy levels to engage with the app. It was also developed in an easy-to-use format to allow for digital literacy.

#### App Usage Recommendations

The app was downloaded by PEARS participants at approximately 16 weeks of gestation and was password-protected so that only participants who were in the intervention group could access it. Participants were encouraged by researchers to use the app every day and they were encouraged to set a recurring alarm on their phones as a reminder. The app had 3 main sections: a *home* page, an *about* page, and a *database of low GI meal ideas and recipes* developed by a research dietician. The home page contained a “Tip of the Day,” which gave a piece of general advice for pregnancy and an “Exercise of the Day” and “Meal of the Day,” which supported the low-GI diet and exercise advice given at the education session. The *about* page contained additional information about the PEARS study and physical activity in pregnancy. Participant app use was recorded by the app’s software from the baseline visit to the woman’s delivery date. App usage data were downloaded and total instances of app use per person were calculated. An “instance” of use of the app was defined as a 15-minute period in which the app was opened once or more.

#### Participant Feedback Questionnaires

Participants were included if they returned a completed feedback questionnaire on intervention acceptability (Multimedia Appendix 1) at 34 weeks of gestation, which assessed compliance with and acceptability of each aspect of the intervention using a Likert-type scale or questions where an option was given to “choose all that apply.” The dietary component of the questionnaire was based on a questionnaire used in the ROLO study [16], which was originally based on questions used in a low GI intervention study by Moses et al [23]. The physical activity questions were based on the Motives for Physical Activity Measure questionnaire [24]. Upon completion of the intervention, a smartphone app acceptability questionnaire, which assessed the enjoyment and acceptability of the app, was completed by the participants (Multimedia Appendix 2). This questionnaire was self-designed, as at the time of study development, there were no existing validated questionnaires examining the use of smartphone apps to deliver a lifestyle intervention during pregnancy.

### Statistical Analyses

Statistical analyses of quantitative data were carried out using SPSS Statistics v.20 (IBM Corp). Descriptions of maternal characteristics and questionnaire responses were presented as mean (SD) or n (%) for continuous and categorical data, respectively. The normality of the continuous variables was assessed visually using histograms. Bivariate analysis was initially carried out to test for associations between maternal characteristics and responses to individual questions in the 2 questionnaires (independent samples two-tailed *t* tests, Mann-Whitney *U* tests, and chi-square analyses). Results were presented as mean (SD), unless otherwise stated. Variables that were significantly associated in bivariate analysis were further analyzed using logistic regression, controlling for age, BMI, socioeconomic status, education level, and parity. The analysis for each question was conducted separately. Mann-Whitney *U* tests were carried out to test for associations between total instances of app use (objectively obtained from the app) and app acceptability (based on questionnaire responses). Where significant associations were obtained, logistic regression analysis was carried out, controlling for smoking, age, socioeconomic status, education level, BMI, parity, and ethnicity.

### Qualitative Interviews

A subset of the PEARS cohort was invited to partake in semistructured one-on-one interviews, in which participants were asked to describe their experiences of the PEARS study and app. A subset of 30 women was to be purposely sampled to recruit a balance of nulliparous (15/30, 50%) and multiparous (15/30, 50%) women who had either obtained a third-level qualification (15/30, 50%) or whose highest education achievement was second-level education or below (15/30, 50%). The interview occurred at 34 weeks of gestation. The interviews were voice-recorded and transcribed by a commercial transcription service. The transcribed interviews were anonymized and thematically analyzed. Participants were assigned pseudonyms for reporting purposes.

## Results

### Maternal Characteristics and Responses to Questionnaires

Of the 278 participants in the intervention group, 149 participants completed the PEARS study feedback form (53.6% response rate) and 123 completed the Smartphone App Evaluation Questionnaire (44.2% response rate). There were no differences in the maternal demographics between those who responded to the questionnaires and those who did not (Multimedia Appendix 3). The characteristics of the respondents are outlined below (Table 1). Responses to both questionnaires are presented in Table 2.

**Table 1:** Characteristics of the participants who completed the questionnaires.

Characteristic	PEARS^a^ study feedback form			Smartphone app evaluation questionnaire	
	n	Values	n		Values
Age (years), mean (SD)	147	32.84 (4.61)	123		32.78 (4.45)
Early-pregnancy weight (kg), mean (SD)	149	78.61 (10.67)	123		78.99 (10.99)
Early-pregnancy BMI (kg/m²), mean (SD)	149	29.21 (3.32)	123		29.11(3.28)
Overweight (BMI 25-29.9 kg/m²), n (%)	149	104 (69.8)	123		90 (73.2)
Obese (BMI ≥30 kg/m²), n (%)	149	45 (30.2)	123		33 (26.8)
Neighborhood deprivation index (Pobal Haase-Pratschke index), mean (SD)	149	5.46 (11.22)	123		5.4 (11.31)
Advantaged, n (%)^b^	149	105 (70.5)	123		84 (68.3)
Disadvantaged, n (%)^c^	149	44 (29.5)	123		39(31.7)
Achieved third-level education, n (%)	145	88 (60.7)	107		63 (58.9)
Smoking in early pregnancy, n (%)	137	8 (5.8)	108		3 (2.8)
Parity, median (IQR)	147	0 (0-1)	122		1 (0-1)
Multiparous, n (%)	147	73 (49.7)	122		62 (50.8)
White-Irish ethnicity, n (%)	148	123 (83.1)	121		100 (82.6)

^a^PEARS: pregnancy, exercise, and nutrition research study with smartphone app support.

^b^Advantaged is indicative of a Pobal Haase-Pratschke index >0.

^c^Disadvantaged is indicative of a Pobal Haase-Pratschke index ≤0.

**Table 2:** Data of the responses on the acceptability of the intervention and smartphone app.

Question		Answer	Values, n (%)
**Intervention acceptability**			
	I have followed the recommended diet (n=153)	Compliant (always or mostly)	101 (67.8)
	It was easy to follow the diet recommended during the study (n=148)	Agree^a^	98 (68.5)
	I enjoyed the dietary changes I made (n=148)	Agree	106 (74.1)
	The changes I made did not increase my weekly grocery bill (n=148)	Agree	110 (76.9)
	My family was happy with the changes I made to my diet (n=148)	Agree	98 (68.5)
	I felt I had enough energy while on the diet (n=149)	Agree	113 (78.5)
	I enjoyed eating a wide variety of foods in my eating plan (n=148)	Agree	118 (82.5)
	I followed the exercise prescription (n=153)	Compliant (regularly)	67 (45.3)
	**I adhered to the exercise that was prescribed to me because** **(Choose all that apply) (n=141)**		
		I was told to by the research team	28 (19.9)
		I knew it was beneficial for me and the pregnancy	123 (87.2)
		I was influenced by the app and other social media	8 (5.7)
		I wanted to feel better about myself	67 (47.5)
		It was unavoidable	15 (10.6)
		I had good support from family and friends	29 (20.6)
	**I was unable to perform exercise because of the following** **(Choose all that apply) (n=83)**		
		Lack of time	46 (55)
		Lack of facilities	1 (1)
		Lack of support	1 (1)
		Weather	31 (37)
		Prohibition by family, friends, doctors	11 (13)
		Didn't care to do it	10 (12)
		Lack of guidance by research team	0 (0)
		Lack of understanding of the exercise	0 (0)
		Worried in case it wasn't safe in pregnancy	7 (9)
		I felt it was ineffective and pointless	0 (0)
**App acceptability**			
	The PEARS^b^ app was enjoyable to use (n=120)		96 (80.0)
	The PEARS app was easy to use (n=119)		116 (97.5)
	The PEARS app was written in language that was easy to understand (n=120)		120 (100.0)
	The PEARS app was attractively presented (n=123)		113 (91.9)
	The PEARS app was graphically helpful (n=118)		99 (83.9)
	The PEARS app was useful (n=122)		112 (91.8)
	The PEARS app made me think about my diet (n=120)		107 (89.2)
	**I found the “Tip of the Day” function**		
		Useful (n=120)	103 (85.8)
		Practical (n=120)	96 (80.0)
		Motivating (n=118)	87 (73.7)
		Motivated me to eat well (n=118)	87 (73.7)
		Motivated me to be active (n=118)	79 (66.9)
		Was helpful in planning meals (n=118)	71 (60.2)
	**I found the “Exercise of the Day” function**		
		Useful (n=121)	91 (75.2)
		Practical (n=119)	85 (71.4)
		Motivating (n=120)	83 (69.2)
	**I found the “Meal of the Day” function**		
		Useful (n=122)	99 (81.1)
		Practical (n=118)	90 (76.3)
		Motivating (n=120)	88 (73.3)
		Agree that the meals on the app were no more expensive than meals I made before I began the study (n=121)	89 (73.6)
	**There was () detail provided on the app (n=122)**		
		Too little	28 (23.0)
		Enough	97 (79.5)
		Too much	0 (0.0)
	**Which of the following enticed you to use the app regularly (Choose all that apply) (n=123)**		
		Looks good	14 (11.4)
		Easy to use	76 (61.8)
		Readily available	67 (54.5)
		Exactly what I need	12 (9.8)
		Answers my questions on diet and exercise	26 (21.1)
		Meal of the Day function	49 (39.8)
		Exercise of the Day function	23 (18.7)
		Tip of the Day function	49 (39.8)
	How many times per week did you use the app? (n=123)	Regularly	79 (64.2)
	Would you recommend this app to a friend? (n=123)	Yes	116 (94.3)
	Would you use this app if you were pregnant again? (n=123)	Yes	115 (93.5)

^a^Agree: the sum of the responses “agree” and “strongly agree.”

^b^PEARS: pregnancy, exercise, and nutrition research study with smartphone app support.

### App Usage

The median weeks of app use among participants was 20 (IQR 11-24) weeks. Over this time period, the median of total instances of app use for participants was 24 (IQR 8-72). The median for instances of app use per week among participants was 1.75 (IQR 0.71-3.49).

### Bivariate Analysis

#### Intervention Acceptability

The mean neighborhood deprivation scores in the group who found the recommended diet easy to follow were significantly lower than those in the group who did not (4.46 [SD 11.25] vs 8.58 [SD 10.95], respectively; *P*=.04). Those who did not achieve tertiary education were more likely to agree that they enjoyed the dietary changes than those who achieved tertiary education (48/54, 89% (SD 32%) vs 55/86, 64% (SD 48%), respectively; *P=*.002). Those who felt that dietary changes did not increase their grocery bill were older than those who did (33.42 [SD 4.68] years vs 31.45 [SD 3.92] years, respectively; *P*=.03). Self-reported compliance with the diet, compliance with the exercise prescription, finding the diet easy to follow, family satisfaction with the changes, satisfaction with energy levels while on the diet, and enjoyment of a variety of foods were not associated with maternal characteristics.

#### Smartphone App Acceptability

Acceptability of most aspects of the app did not differ by maternal characteristics. Questions that were answered differently by subgroups of women are detailed below. The group who agreed that the graphics on the app were helpful scored lower for affluence on the HP deprivation index than those who disagreed (4.52 [SD 11.3] vs 10.29 [SD 10.8], respectively; *P*=.03). Those from disadvantaged neighborhoods were more likely to find the graphics helpful than those in advantaged neighborhoods (35/37, 95% [SD 23%] vs 62/81, 77% [SD 43%], respectively; *P=*.03). Those who found the “Tip of the Day” useful had a lower HP index than those who did not (4.55 [SD 11.56] vs 10.33 [SD 7.97], respectively; *P*=.01). Those who agreed that the tip was helpful in planning meals had a lower HP index (2.87 [SD 11.43] vs 9.23 [SD 9.92], respectively; *P*=.002) and were more likely to select this response if from a disadvantaged neighborhood compared to those from an advantaged neighborhood (29/37, 78% [SD 42%] vs 41/81, 51% [SD 50%], respectively; *P=*.008). The group who found the “Exercise of the Day” practical had a lower HP index than those who disagreed (3.83 [SD 12.01] vs 10.11 [SD 7.8], respectively; *P*=.001) and those who were from disadvantaged neighborhoods were more likely to agree than those from advantaged groups (32/37, 86% [SD 35%] vs 52/82, 63% [SD 49%], respectively; *P*=.02). The group who agreed that the “Exercise of the Day” was motivating had a higher median BMI than the group that disagreed (28.74 [IQR 26.7-31.49] vs 27.49 [IQR 26.33-29.22], respectively; *P*=.04).

Those who found the “Meal of the Day” useful had a higher median BMI (28.71 [IQR 26.71-31.39) vs 26.74 [IQR 26.18-29.31], respectively; *P*=.02) than those who disagreed. Those from a disadvantaged neighborhood were more likely to agree than those from advantaged neighborhoods (36/39, 92% [SD 27%] vs 61/83, 74% [SD 44%], respectively; *P*=.03). Participants with an obese BMI were more likely to agree that the “Meal of the Day” was motivating than those with an overweight BMI (29/32, 91% [SD 30%] vs 58/87, 67% [SD 47%], respectively; *P*=.02). The group who agreed that the meals on the app were affordable had a lower HP index (4.28 [SD 11.8] vs 8.94 [SD 8.88], respectively; *P*=.02). The group who thought there was enough detail had lower HP index than those who thought there was too little detail (3.87 [SD 10.83] vs 10.32 [SD 11.84], respectively; *P*=.008). Those who achieved less than third-level education were more likely to think there was enough information provided on the app (39/44, 89% [SD 32%] vs 43/62, 69% [SD 47%], respectively; *P*=.04). Younger women were more likely to recommend this app to a friend (32.51 [SD 4.49] vs 35.77 [SD 2.64], respectively; *P*=.03) as well as those with a lower HP index (4.77 [SD 11.18] vs 12.5 [SD 10.93], respectively; *P*=.04). App use was significantly associated with enjoyment of the app, finding the graphics helpful, and acceptability of the “Tip of the day,” “Meal of the Day,” and “Exercise of the Day” sections of the app (Table 3).

**Table 3:** Comparison of the objectively measured total app usage instances according to app acceptability by using Mann-Whitney *U* tests.

Question	Agree^a^, median (IQR)	Disagree^b^, median (IQR)	*P* value
The PEARS^c^ app was enjoyable to use (n=114)	28.5 (12-80.75)	11 (6-25)	.006
The PEARS app was easy to use (n=113)	24 (8-73)	10 (4-^d^)	.31
The PEARS app was straightforward to follow (n=113)	25 (8-73)	10 (4- ^d^)	.32
I found the graphics helpful (n=112)	28 (8.5-73)	14 (5.25-42.5)	.04
The PEARS app was useful (n=116)	24 (9.25-72.5)	12 (4.5-68.25)	.28
I found the “Tip of the Day” function useful (n=114)	21 (8-69.5)	28 (6.5-82)	.88
I found the “Tip of the Day” function practical (n=114)	24 (7.5-69.5)	20 (8.5-68)	.88
I found the “Tip of the Day” function motivating (n=114)	26 (8.5-78.25)	17.5 (7-48.75)	.23
I found the “Tip of the Day” function motivated me to eat well (n=112)	28.5 (10.5-83)	15 (6.25-43.75)	.02
I found the “Tip of the Day” function motivated me to be active (n=112)	28.5 (10-84.25)	19 (6.75-41.5)	.06
I found the “Tip of the Day” function was helpful in planning meals (n=113)	24 (10-84.25)	20.5 (7.75-73.5)	.97
I found the “Exercise of the Day” function useful (n=115)	25 (7.25-80)	16 (8-56)	.26
I found the “Exercise of the Day” function practical (n=113)	28 (12-83)	15.5 (6-38)	.02
I found the “Exercise of the Day” function motivating (n=114)	28 (11-76.5)	19 (6-52.5)	.14
I found the “Meal of the Day” function useful (n=116)	25 (7.25-78.25)	20 (8-48.75)	.52
I found the “Meal of the Day” function practical (n=112)	28 (10-81.5)	17 (6-28)	.03
I found the “Meal of the Day” function motivating (n=113)	28 (10-80)	17.5 (6-48.25)	.12
The meals on the app were no more expensive than meals I made before I began the study (n=115)	26 (12-74)	19.5 (6-60.25)	.23
There was enough detail provided on the app (n=116)	24 (10.5-70.25)	23.5 (6.25-85.5)	.74
Would you recommend this app to a friend? (n=117)	24 (8-73)	21.5 (5.5-60.75)	.44
Would you use this app if you were pregnant again? (n=117)	25 (8.75-73)	10 (4-41)	.11

^a^Agree: the sum of the responses “agree” and “strongly agree.”

^b^Disagree: the sum of the responses “disagree” and “strongly disagree.”

^c^PEARS: pregnancy, exercise, and nutrition research study with smartphone app support.

^d^75th percentile was not available in the analysis output.

### Multivariate Analysis

In terms of intervention acceptability, in multivariate analysis, enjoyment of the dietary changes undertaken as part of the intervention was significantly associated with a less than third-level education, independent of the effect of confounding factors (Table 4). When further analyzing the app acceptability responses, disadvantaged neighborhood deprivation was significantly associated with agreeing that there is enough detail provided on the app (*P*=.009). There was also a significantly positive association between finding the “Exercise of the Day” motivating and BMI, independent of the effects of confounding factors (*P*=.048) (Table 5). App usage was significantly higher among those who found that the “Tip of the Day” motivated them to eat well (*P*=.03) and who found the “Exercise of the Day” practical (*P*=.004) (Table 6).

**Table 4:** Multivariate analysis of the association between intervention acceptability and maternal characteristics.^a^

Question	Variable	B^b^ coefficient	*P* value	Odds ratio (95% CI)	*P* value^c^
It was easy to follow the diet recommended during the study (n=136)	Pobal Haase-Pratschke index	–0.039	.04	0.962 (0.926- 0.999)	.14
I enjoyed the dietary changes I made (n=137)	Education (achieved third-level)	–1.386	.007	0.25 (0.092-0.683)	.04
The changes I made did not increase my weekly grocery bill (n=136)	Age (years)	0.125	.02	1.133 (1.017-1.262)	.06

^a^Controlling for BMI, age, education level, Pobal Haase-Pratschke index, and parity in a logistic regression model. Only variables that were significant in their respective bivariate models are shown.

^b^Coefficient for the constant in the null model.

^c^Refers to the *P* values in the omnibus test of model coefficients.

**Table 5:** Multivariate analysis of the association between app acceptability and maternal characteristics.^a^

Question	Variable	B^b^ coefficient	*P* value	Odds ratio (95% CI)	*P* value^c^	
I found the graphics helpful (n=99)	Pobal Haase-Pratschke index	–0.082	.01	0.922 (0.865-0.982)	.08	
I think the app needs more pictures (n=98)	Age	0.087	.11	1.091 (0.98-1.214)	.48	
**The PEARS^d^ app is as good, if not better, than other apps available for pregnancy (n=100)**						.05
	Age	–0.115	.04	0.891 (0.801-0.992)		
	Education (achieved third-level)	0.579	.22	1.785 (0.702-4.538)		
I found the “Tip of the Day” function useful (n=101)	Pobal Haase-Pratschke index	–0.046	.15	0.955 (0.896-1.017)	.28	
I found the “Tip of the Day” function was helpful in planning meals (n=99)	Pobal Haase-Pratschke index	–0.062	.009	0.94 (0.898-0.984)	.06	
**I found the “Exercise of the Day” function useful (n=102)**						.16
	Pobal Haase-Pratschke index	–0.047	.06	0.954 (0.91-1.001)		
	BMI	0.145	.11	1.156 (0.969-1.38)		
I found the “Exercise of the Day” function practical (n=100)	Pobal Haase-Pratschke index	–0.050	.04	0.951 (0.907-0.998)	.08	
I found the “Exercise of the Day” function motivating (n=101)	BMI	0.172	.048	1.188 (1.001-1.409)	.03	
**I found the “Meal of the Day” function useful (n=103)**						.06
	Pobal Haase-Pratschke index	–0.046	.10	0.955 (0.904-1.008)		
	BMI	0.224	.053	1.251 (0.997-1.57)		
I found the “Meal of the Day” function practical (n=98)	Smoking	–22.053	>.99	0 (0)	.09	
I found the “Meal of the Day” function motivating (n=100)	BMI	0.204	.02	1.226 (1.029-1.461)	.16	
The meals on the app were helpful for preparing breakfast (n=101)	Age	–0.108	.07	0.898 (0.799-1.009)	.41	
The meals on the app were no more expensive than meals I made before I began the study (n=102)	Pobal Haase-Pratschke index	–0.050	.04	0.951 (0.906-0.998)	.08	
**There was enough detail provided on the app (n=103)**						.01
	Pobal Haase-Pratschke index	–0.082	.009	0.921 (0.866-0.979)		
	Education (achieved third level)	0.762	.24	2.142 (0.601-7.63)		
**Would you recommend this app to a friend? (n=104)**						.15
	Age	–0.228	.04	0.796 (0.642-0.988)		
	Pobal Haase-Pratschke index	–0.048	.23	0.953 (0.88-1.032)		

^a^Controlling for BMI, age, education level, Pobal Haase-Pratschke index, and parity in a logistic regression model. Only variables that were significant in their respective bivariate models are displayed above.

^b^Coefficient for the constant in the null model.

^c^Refers to the *P* values in the omnibus test of model coefficients.

^d^PEARS: pregnancy, exercise, and nutrition research study with smartphone app support.

**Table 6:** Association between app acceptability and app instances of use (obtained from the app’s software).^a^

Question	B^b^ coefficient	*P* value	Odds ratio (95% CI)	*P* value^c^
The PEARS^d^ app was enjoyable to use (n=114)	0.026	.04	1.027 (1.002-1.052)	.10
I found the graphics helpful (n=112)	0.018	.07	1.018 (0.998-1.038)	.04
I found the “Tip of the Day” function motivated me to eat well (n=112)	0.021	.02	1.021 (1.003-1.039)	.03
I found the “Exercise of the Day” function practical (n=113)	0.020	.009	1.02 (1.005-1.035)	.004
I found the “Meal of the Day” function practical (n=112)	0.011	.13	1.011 (0.997-1.025)	.06
I found the “Meal of the Day” function appetizing (n=113)	0.012	.07	1.012 (0.999-1.025)	.42
The meals on the app were helpful for preparing dinner (n=116)	0.007	.34	1.007 (0.993-1.02)	.37

^a^Controlling for smoking, age, Pobal Haase-Pratschke index, education level, BMI, parity, and ethnicity in a logistic regression model. Only variables that were significant in their respective bivariate models are displayed above.

^b^Coefficient for the constant in the null model.

^c^Refers to the *P* values in the omnibus test of model coefficients.

^d^PEARS: pregnancy, exercise, and nutrition research study with smartphone app support.

### Qualitative Interviews

Interviews were conducted with 28 PEARS participants whose characteristics are outlined in Table S1 of Multimedia Appendix 4. The majority of the participants were very satisfied with the content of the PEARS study.

…Like truthfully, it all worked for me. Like I had the app to give me ideas and I had you and (the PEARS study doctor) if I needed supports. So for me, there really isn’t anything I would change. And I’m not saying that to be nice. I honestly, it was fine, it was helpful for me and it motivated me. But it never took over, and that’s what I liked about it.

Sheila, intervention, 39 years old, nulliparous, BMI 28, White Irish, second-level education

### Diet Intervention

Most women made positive changes to their diet since participation in the PEARS study. The majority of women found the dietary changes easy to make and reported on average that they complied with the recommendations approximately 80% of the time.

…No it wasn’t actually that difficult, when I got into the swing of it. Now I’d say the first couple of weeks, I was very conscious of what I was doing. And then it was sure nothing.

Nicole, intervention, 36 years old, parity 4, BMI 25, White Irish, third-level education

The wide variety of food options when following a low-GI diet and the idea of swapping foods rather than eliminating them made the recommendations more acceptable and sustainable for participants. However, there were varying levels of acceptability of the financial cost of following a low-GI diet. Some participants reported that healthy foods were more expensive but that this was balanced by the avoidance of certain snack foods.

…Not really I was buying a lot more fruit, so that was a bit more expensive and the kind of salad stuff and the veg kind of would bring the shopping bill up a bit, but I mean I think it was worth it like and as I said I cut out buying crisps and chocolate covered biscuits and stuff so then it was coming down as well.

Orla, intervention, 30 years old, parity 1, BMI 25, White Irish, third-level education

### Exercise Intervention

There was a substantially less positive change in physical activity behavior during pregnancy. Time constraints were cited as the most common barrier to exercise.

…If I had time, [if] it was my first pregnancy, I’d probably have a lot more time, more effort.

Lianne, intervention, 37 years old, parity 1, BMI 25, White Irish, second-level education

However, the encouragement of physical activity made women with low self-efficacy perceive physical activity as accessible.

…Even with the exercise say, with the 30 minutes a day, when you told me oh you can split that up over three 10 minutes sure fabulous, like who can’t fit in three 10 minutes a day?

Sally, intervention, 31 years old, nulliparous, BMI 26, Irish, third-level education

### Smartphone App Intervention

Overall, the women appeared very satisfied with the app and the majority agreed that it played a role in their adherence to the PEARS dietary recommendations. Participants valued the reliability of the PEARS app as the content was created and approved by a multidisciplinary team.

…I know with your app that’s 100 per cent guaranteed - it has the backup, it has all the right information.

Caoimhe, intervention, 34 years old, parity 3, BMI 34, White Irish, second-level education

The “Tip of the Day” was the strongest enticement for interview participants to access the app.

…I probably would go into the app purposely on a daily basis to read the ‘Tip of the Day’.

Laura, intervention, 26 years old, nulliparous, BMI 28, Irish, third-level education

Some interview participants reported a decline in their use of their app throughout the intervention. This was particularly evident among women who had achieved tertiary education due to perceived confidence in their cooking abilities and knowledge.

…I think it’s because I thought I was familiar with the material. I think it’s because our meeting kind of armed me and I felt like okay well I know what I’m not supposed to do and I know what I can do, so just do that and then I didn’t check in with it you know. … I thought that the meals I was having already would probably conform to the diet.

Kelly, intervention, 34 years old, nulliparous, BMI 26, White Irish, third-level education

## Discussion

### Principal Results

The PEARS diet and lifestyle intervention with smartphone app support was accepted by a pregnant cohort who were overweight or obese and greater acceptability was associated with lower education level. The use of a smartphone app in the intervention was deemed highly acceptable and aspects of acceptability were associated with lower socioeconomic status and higher BMI. Enjoyment of certain sections of the app was associated with increased use, as measured by the app’s software.

### Mixed Lifestyle Intervention Acceptability

The acceptability of mixed lifestyle interventions has been reported in previous studies among similar cohorts of pregnant women [4,7,19]. Participants of PEARS reported to be compliant with the dietary aspect of the intervention and found a low-GI diet enjoyable and easy to follow. In a previous study of a low-GI diet in pregnancy (ROLO Study), which did not incorporate an exercise or mHealth component, acceptability of the diet was also high [20]. Similar results were found in both the studies; in the ROLO study, 68% found the low-GI diet easy to follow and 65% found it enjoyable, while in the PEARS study, 69% found it easy to follow and 74% found it enjoyable [20]. The use of smartphone app technology as part of the PEARS intervention was deemed acceptable by the cohort of pregnant women who were overweight or obese, as reported in both the quantitative and qualitative analyses. High acceptability of the use of mHealth was also reported in other mixed lifestyle interventions among pregnant women with a high BMI [4,6,7] and highlights mHealth as a positive way for health care professionals to communicate reliable health and lifestyle information to their patients. Having a higher BMI was associated with acceptability of the “Exercise of the Day” subsection of the app. A study among college-aged women has shown that high BMI is associated with experiences of weight stigma, which is associated with exercise avoidance [25]. As those with high BMI particularly enjoyed the exercise portion of the PEARS app, the app may have been successful in overcoming this barrier and encouraging the enjoyment of exercise.

### Engagement With Women of Low Socioeconomic Status

Low education level is often associated with poor dietary habits and poor response to dietary interventions [26,27] and has been associated with gestational weight gain outside the recommendations in pregnancy [28]. Contrastingly, this study reports higher acceptability of a dietary intervention among participants with lower education level, a group that may have been required to make the greatest change to their preintervention diet to comply with the recommended diet. The PEARS dietary intervention is accepted by a group with higher likelihood of having poor dietary habits before pregnancy and who are more likely to exceed the recommended gestational weight gain [26-28]. Therefore, this group may have the most potential to benefit from a dietary intervention. The qualitative analysis suggests that women with higher educational attainment more commonly discontinued their use of the app throughout the course of the intervention, as they felt confident in their knowledge and cooking abilities. A smartphone app with recipes and meal ideas may be a useful tool for those with less confidence in their cooking abilities and nutrition knowledge. This study-specific smartphone app was deemed easy to use and to understand by all participants, including those from hard-to-reach subgroups of the population. Low socioeconomic status was associated with acceptability of the level of detail provided on the app. The addition of mHealth technology to the PEARS study enabled women with all levels of literacy and previous nutrition knowledge to engage with the intervention. If used in future studies or for public health purposes, smartphone apps may be adapted to the literacy levels of the target population to assist in improving health outcomes. Our findings support mHealth apps as a tool that may help in bridging the socioeconomic health gap, which exists in Ireland, by providing an adequate and accessible source of additional health care information [9].

### Clinical Implications

The “Tip of the Day” function was reported to be the most useful subsection of the app, as reported in the app questionnaire. Results from the qualitative interviews support this finding, as participants reported that this feature was their highest motivator in accessing the app. This section provided a small piece of information in a concise manner. This finding is highly relevant to the current health care climate in Ireland, which is introducing the concept of “Making Every Contact Count” (MECC). It is a health behavior change framework that will be implemented to take advantage of the interactions health care professionals have with patients in order to send positive health care messages, which ultimately aim to reduce the incidence of chronic disease [28]. The basic level of its implementation is giving patients brief healthy lifestyle advice when the opportunity arises. The MECC framework plans to incorporate mHealth as a tool to communicate with both patients and staff. A smartphone app that contains a feature such as “Tip of the Day,” which delivers brief diet and exercise advice may be of use in such a behavior change framework, as in this study, it was found to be acceptable in a pregnant cohort at a higher risk of developing GDM. mHealth also has the potential to support current dietetic practice as part of individual patient counselling or to deliver novel and convenient nutrition information to hard-to-reach patient groups.

### Future of Health Care Apps

Most participants stated they would use the app in pregnancy again and recommend it to others. Of the entire PEARS cohort of 565 participants, 379 (67.1%) participants in early pregnancy reported using a pregnancy app as a source of information (unpublished data from the PEARS study). Although this figure may encompass a wide variety of pregnancy apps (eg, gestation calculators, pregnancy tracking, health information), it is known that there is a lack of regulation across all types of commercial pregnancy apps [11]. This highlights the demand for the development of suitable apps, designed and approved by health care professionals, particularly pregnancy apps that provide diet and lifestyle advice. Access to reliable diets and lifestyle information in a pregnancy app also appears to be important to pregnant women, as interview participants valued that the PEARS app contained reliable information, approved by health care professionals. App usage was higher among those who found the “Tip of the Day” motivating and those who found the “Exercise of the Day” practical. This demonstrates that the delivery of information in a concise format and practical information and advice were valuable to participants. Features that make the app clear and non–time-consuming would be important aspects to consider if creating future mHealth apps for similar populations.

### Strengths and Limitations

The strengths of this study include the large population size and the availability of app usage data in order to verify participant app use. The cohort studied is well-characterized and a large amount of data was collected on maternal characteristics in early pregnancy. This was also a representative sample of the PEARS study (Multimedia Appendix 3). This is the first pregnancy study to report on the acceptability of an app used to deliver healthy lifestyle information, thus adding to this field. The availability of both quantitative and qualitative data was also a strength of this research.

A limitation of this study was that compliance and acceptability of both the intervention and the app were self-reported, thereby possibly introducing incorrect reporting. Compliance with recommended app use could be verified using the app’s software; however, diet and exercise compliance were not verified by any other measurements. Another limitation was that the questions in the questionnaires were not open-ended. Participants were limited to choosing from a list of responses that were preselected by the research team, which may have influenced their true opinion. However, this is also a strength as it allows for statistical analysis. These findings are specific to a pregnant cohort with a BMI ≥25 kg/m², which is not representative of the BMI of a general pregnant population. However, given that half of pregnant women in Ireland and other Western populations have a BMI in the overweight or obese categories [29,30], our findings are relevant for all clinicians working in the antenatal setting. As individuals who volunteer for research studies typically have higher education and are older than those who decline participation [31], the generalizability of our findings to a broader population group may be affected. Responses were also not received from all participants of the intervention group for unknown reasons; however, there were no differences in the maternal demographics between respondents and nonrespondents. An important limitation to this research was 174 (9.4%) women who were assessed for eligibility for the PEARS study (n=1858) did not own a smartphone and were therefore ineligible to participate in the intervention. Although results have demonstrated that aspects of the app showed higher acceptability among participants from lower socioeconomic backgrounds, the exclusion criteria of the study may have excluded the most hard-to-reach women, who may not be in possession of a smartphone. It is also evident from the app usage data that the app was not used as often as recommended by researchers. Results demonstrate that app usage was associated with acceptability of certain aspects of the app; usage was higher among participants who enjoyed “Exercise of the Day” and “Tip of the Day.” Therefore, if the app had been used more regularly, perhaps the participants’ acceptability may have differed.

### Conclusions

A low-GI diet and physical activity intervention with smartphone app support was accepted by a population of pregnant women who were overweight or obese. The diet was considered enjoyable and easy to follow, and compliance was high. The acceptability of the smartphone app was also high. Although the smartphone app used in the PEARS study was not targeted at a population of specific socioeconomic status or education level, results demonstrate that aspects of the app were particularly accepted by these hard-to-reach groups. The app appealed to those who had a higher BMI and were therefore more at risk of developing GDM. When developing a future app for a similar population, the socioeconomic and educational characteristics of the population should be considered in order to adapt the level of detail and presentation of information accordingly.

## Appendix

Multimedia Appendix 1 Intervention acceptability questionnaire.

Multimedia Appendix 2 App acceptability questionnaire.

Multimedia Appendix 3 Comparison of maternal demographics between questionnaire respondents and nonrespondents.

Multimedia Appendix 4 Characteristics of the respondents to the qualitative interviews.

## Abbreviations

GDM: gestational diabetes mellitus
GI: glycemic index
HP: Haase-Pratschke
MECC: Making Every Contact Count
mHealth: mobile health
PEARS: pregnancy, exercise, and nutrition research study with smartphone app support
