# Supplementary material for: Acceptability of the Pregnancy, Exercise, and Nutrition Research Study With Smartphone App Support (PEARS) and the Use of Mobile Health in a Mixed Lifestyle Intervention by Pregnant Obese and Overweight Women: Secondary Analysis of a Randomized Controlled Trial
Source: JMIR Mhealth Uhealth. 2021 May 12;9(5):e17189. doi: 10.2196/17189 (PMC8156124; doi:10.2196/17189)
Supplement: Multimedia Appendix 1 [file mhealth_v9i5e17189_app1.pdf]

PEARs Study Feedback Form

Name: ________________________ Hospital number: _______________
Date: _________________________ Study ID: ______________________

Thank you for taking part in the PEARs study in the National Maternity Hospital.

PART 1: NUTRITION

Please tick the box beside the phrase which best describes **how closely you followed the diet** advised in this study:

Q1. I have followed the recommended diet

1) all of the time ☐
2) most of the time ☐
3) sometimes ☐
4) rarely ☐
5) none of the time ☐

Why do you think this was?
(i.e. what made it easy or difficult to follow the low GI diet?)
____
____

Any other comments:
(we value your opinions on any aspect of this study)
____
____

Q2. Please indicate how much you agree or disagree with each of these statements by marking a tick ☑ in the appropriate box:

	**Strongly agree**	**Agree**	**Neither agree or disagree**	**Disagree**	**Strongly disagree**
**It was easy to follow the diet recommended during the study**					
**I enjoyed the dietary changes I made**					
**The changes I made did not increase my weekly grocery bill**					
**My family was happy with the changes I made to my diet**					
**I felt I had enough energy while on the diet**					
**I enjoyed eating a wide variety of foods in my eating plan**					

PART 2: PHYSICAL ACTIVITY

Please tick the box beside the phrase which best describes **how closely you followed the physical activity plan** advised in this study:

Q3. I followed the exercise prescription (please tick one)

1) Regularly ☐
2) Sometimes ☐
3) Occasionally ☐
4) Started regularly but didn't continue ☐
5) None of the time ☐

Q4. I performed exercise (please tick one)

1) 5-7 days per week ☐
2) 3-5 days per week ☐
3) 1 day per week ☐
4) Other ☐
5) Uncertain ☐

Q5. Type of exercise performed (please tick all that apply)

1) Walking ☐
2) Swimming ☐
3) Jogging ☐
4) Aerobics ☐
5) Strength training (weights) ☐
6) Other ☐

Q 6. I adhered to the exercise that was prescribed to me because (please tick all that apply)

1) I was told to by the research team ☐
2) I knew it was beneficial to me and for the pregnancy ☐
3) I was influenced by the app and other social media ☐
4) I wanted to feel better about myself ☐
5) It was unavoidable ☐
6) I had good support from family and friends ☐

Q7. I was unable to perform exercise because of the following (Please tick all that apply)

1) Lack of time ☐
2) Lack of facilities ☐
3) Lack of support ☐
4) Weather ☐

5) Prohibition by family, friends, doctors ☐
6) Didn't care to do it ☐
7) Lack of guidance by research team ☐
8) Lack of understanding of the exercise ☐
9) Worried in case it wasn't safe in pregnancy ☐
10) I felt it was ineffective and pointless ☐

Q8. I previously engaged in regular physical activity outside of pregnancy but stopped it because

1) Lack of time ☐
2) Lack of facilities ☐
3) Lack of support ☐
4) Weather ☐
5) Prohibition by family, friends, doctors ☐
6) Didn't care to do it ☐
7) Lack of guidance by medical professionals ☐
8) Lack of understanding of the exercise ☐
9) I felt it was ineffective and pointless ☐

Thank you for your time
