# Supplementary material for: Acceptability of the Pregnancy, Exercise, and Nutrition Research Study With Smartphone App Support (PEARS) and the Use of Mobile Health in a Mixed Lifestyle Intervention by Pregnant Obese and Overweight Women: Secondary Analysis of a Randomized Controlled Trial
Source: JMIR Mhealth Uhealth. 2021 May 12;9(5):e17189. doi: 10.2196/17189 (PMC8156124; doi:10.2196/17189)
Supplement: Multimedia Appendix 2 [file mhealth_v9i5e17189_app2.pdf]

PEARs Study

Smart Phone App Evaluation Questionnaire

~ Week 34 ~

**Name:**	**Study ID:**	**Date:**

- Thank you for taking part in the PEARs study. You were part of the study group who received nutrition and exercise advice supported by the PEARs smart phone app.
- We want to know what you thought of the app.
- This will help us improve the app for women who use it in the future.
- As with all of your personal data, your reply will be treated as confidential.

Please indicate your level of agreement with each of the following where (1) is strongly agree and (5) is strongly disagree

Section 1: Ease of Use					
**The PEARs app was…**	**Strongly agree**	**Agree**	**Neutral**	**Disagree**	**Strongly disagree**
- enjoyable to use	1	2	3	4	5
- easy to use	1	2	3	4	5
- straight forward to follow	1	2	3	4	5
- easy to download	1	2	3	4	5
- easy to set up	1	2	3	4	5
- easy to navigate through	1	2	3	4	5
- was written in language that was easy to understand	1	2	3	4	5

Section 2: Presentation					
	**Strongly agree**	**Agree**	**Neutral**	**Disagree**	**Strongly disagree**
The app was attractively presented (looked good)	1	2	3	4	5
I found the graphics (pictures) helpful	1	2	3	4	5
I think the app needs more pictures	1	2	3	4	5

Section 3: Benefit					
**The PEARs app…**	**Strongly agree**	**Agree**	**Neutral**	**Disagree**	**Strongly disagree**
- was useful	1	2	3	4	5
- made me think about my diet	1	2	3	4	5
- is as good, if not better, than other apps available for pregnancy	1	2	3	4	5

Section 4: Sub-sections of the App					
I found the 'Tip of the Day' function…	Strongly agree	Agree	Neutral	Disagree	Strongly disagree
- useful	1	2	3	4	5
- practical	1	2	3	4	5
- motivating	1	2	3	4	5
- motivated me to eat well	1	2	3	4	5
- motivated me to be active	1	2	3	4	5
- was helpful in planning meals	1	2	3	4	5

I found the 'Exercise of the Day' function…	Strongly agree	Agree	Neutral	Disagree	Strongly disagree
- useful	1	2	3	4	5
- practical	1	2	3	4	5
- motivating	1	2	3	4	5

I found the 'Meal of the Day' function…	Strongly agree	Agree	Neutral	Disagree	Strongly disagree
- useful	1	2	3	4	5
- practical	1	2	3	4	5
- motivating	1	2	3	4	5
- appetising	1	2	3	4	5

The meals on the app were…	Strongly agree	Agree	Neutral	Disagree	Strongly disagree
- helpful for preparing breakfast	1	2	3	4	5
- helpful for preparing lunch	1	2	3	4	5
- helpful for preparing dinner	1	2	3	4	5
- helpful for preparing snacks	1	2	3	4	5
- easy to follow	1	2	3	4	5
- no more expensive than meals I made before I began the study	1	2	3	4	5
- time-consuming to follow	1	2	3	4	5

I used the exercise portion of the app	Strongly agree	Agree	Neutral	Disagree	Strongly disagree
- which improved my exercise knowledge	1	2	3	4	5
- which answered all of my questions about exercise in pregnancy	1	2	3	4	5

I used the nutrition/dietary portion of the app	Strongly agree	Agree	Neutral	Disagree	Strongly disagree
- which improved my nutrition knowledge	1	2	3	4	5
- which answered all my questions about the low GI diet in pregnancy	1	2	3	4	5

1. There was ☐Too little / ☐Enough / ☐Too much detail provided on the app

2. Which of the following **enticed you** to use the app regularly (you can tick more than 1):

Looks good	☐
Easy to use	☐
Readily available	☐
Exactly what I need	☐
Answers my questions on diet and exercise	☐
'Meal of the Day' function	☐
'Exercise of the Day' function	☐
'Tip of the Day' function	☐
Other *(please specify: ________________)*	☐
I did not use the app regularly	☐

3. How many times per week do you use the app?

☐> 7 times	☐ 6-7 times	☐ 4-5 times	☐ 1-3 times	☐ 0 times

4. Where did you use the PEARs app mostly?

Home	☐	
Office / work place	☐	
Another location	☐	*(please specify:_______________)*
I didn't use it	☐	

5. What did you learn from this app?

6. Please write down any questions about the low GI diet and exercise that you feel were not answered by the app:

Section 5: Future Use

Would you recommend this app to a friend?	
Would you use this app if you were pregnant again?	
What could improve the educational aspects of the app?	
Did anything about the app confuse you?<br>*(content, layout, design, the point of the app, etc.)*	
What features should we add to improve the app?	

Thank you for your time
