# Supplementary material for: Acceptability of the Pregnancy, Exercise, and Nutrition Research Study With Smartphone App Support (PEARS) and the Use of Mobile Health in a Mixed Lifestyle Intervention by Pregnant Obese and Overweight Women: Secondary Analysis of a Randomized Controlled Trial
Source: JMIR Mhealth Uhealth. 2021 May 12;9(5):e17189. doi: 10.2196/17189 (PMC8156124; doi:10.2196/17189)
Supplement: Multimedia Appendix 3 [file mhealth_v9i5e17189_app3.pdf]

Supplemental Table 1. Comparison of maternal demographics of respondents to the Intervention Acceptability Questionnaire and non-respondents.

**Variable**		**Responded to Intervention Acceptability Questionnaire**			**Did not respond to Intervention Acceptability Questionnaire**			**P**
		**N**			**N**			
**Age (Years)** [a]		147	32.84	(4.61)	91	33.13	(4.61)	0.650
**HP index**[a]		149	5.54	(11.21)	91	5.87	(11.27)	0.828
**Weight (Kg)** [b]		149	76.30	(70.15-85.30)	91	79.50	(72.90-84.60)	0.212
**BMI (Kg/m²)**[b]		149	28.30	(26.71-31.07)	91	28.40	(26.78-31.54)	0.555
**Education**[c]	Achieved third level	136	81	(59.6)	74	40	(54.1)	0.532
	Achieved less than third level	136	55	(40.4)	74	34	(45.9)	
**BMI category**[c]	Overweight	149	104	(69.8)	91	62	(68.1)	0.899
	Obese	149	45	(30.2)	91	29	(31.9)	
**Parity**[c]	Previous children	149	73	(49.0)	91	51	(56.0)	0.354
	No previous children	149	76	(51.0)	91	40	(44.0)	
**HP Category**[c]	Above average	149	105	(70.5)	91	61	(67.0)	0.678
	Below average	149	44	(29.5)	91	30	(33.0)	
**Ethnicity**[c]	White Irish	145	122	(84.1)	85	66	(77.6)	0.292
	Non-White-Irish	145	23	(15.9)	85	19	(22.4)	
**Smoking**[c]	Yes	137	7	(5.1)	73	2	(2.7)	0.653
	No	137	130	(94.9)	73	71	(97.3)	

BMI; Body Mass Index HP; Haase and Pratschke

[a] Continuous parametric data are represented as Mean(Standard Deviation) and were compared using independent samples t-tests

[b] Continuous non-parametric data are represented as Median(Interquartile Range) and were compared using Mann-Whitney U tests.

[c] Categorical data are represented as [N (%)] and were compared using a Chi-squared test.

Supplemental Table 2. Comparison of maternal demographics of respondents to the App Acceptability Questionnaire and non-respondents.

Variable		Responded to App Acceptability Questionnaire			Did not respond to App Acceptability Questionnaire			P
		N			N			
**Age (Years)** [a]		120	32.67	(4.45)	118	33.25	(4.76)	0.333
**HP index**[a]		121	5.13	(11.46)	119	6.21	(10.97)	0.457
**Weight (Kg)** [b]		121	76.20	(70.60-83.70)	119	78.40	(71.20-85.80)	0.547
**BMI (Kg/m²)**[b]		121	28.30	(26.51-30.63)	119	28.44	(26.99-31.60)	0.259
**Education**[c]	Achieved third level	105	61	(58.1)	105	60	(57.1)	1.000
	Achieved less than third level	105	44	(41.9)	105	45	(42.9)	
**BMI category**[c]	Overweight	121	89	(73.6)	119	77	(64.7)	0.179
	Obese	121	32	(26.4)	119	42	(35.3)	
**Parity**[c]	Previous children	121	60	(49.6)	119	64	(53.8)	0.602
	No previous children	121	61	(50.4)	119	55	(46.2)	
**HP Category**[c]	Above average	121	81	(66.9)	119	85	(71.4)	0.540
	Below average	121	40	(33.1)	119	34	(28.6)	
**Ethnicity**[c]	White Irish	117	98	(83.8)	113	90	(79.6)	0.524
	Non-White-Irish	117	19	(16.2)	113	23	(20.4)	
**Smoking**[c]	Yes	106	3	(2.8)	104	6	(5.8)	0.477
	No	106	103	(97.2)	104	98	(94.2)	

BMI; Body Mass Index HP; Haase and Pratschke

[a] Continuous parametric data are represented as Mean(Standard Deviation) and were compared using independent samples t-tests

[b] Continuous non-parametric data are represented as Median(Interquartile Range) and were compared using Mann-Whitney U tests.

[c] Categorical data are represented as [N (%)] and were compared using a Chi-squared test.
