# Supplementary material for: Acceptability of the Pregnancy, Exercise, and Nutrition Research Study With Smartphone App Support (PEARS) and the Use of Mobile Health in a Mixed Lifestyle Intervention by Pregnant Obese and Overweight Women: Secondary Analysis of a Randomized Controlled Trial
Source: JMIR Mhealth Uhealth. 2021 May 12;9(5):e17189. doi: 10.2196/17189 (PMC8156124; doi:10.2196/17189)
Supplement: Multimedia Appendix 4 [file mhealth_v9i5e17189_app4.pdf]

Table S1. Maternal characteristics of the respondents to the qualitative interviews (n=28)

Characteristic	Values
Age (Years), mean (SD)	32.8 (5.0)
Early-pregnancy weight (kg), mean (SD)	77.6 (13.6)
Early-pregnancy BMI (kg/m^2), mean (SD)	28.0 (3.6)
Overweight, n (%)	23 (82.1)
Obese, n (%)	5 (17.9)
Achieved third level education, n (%)	15 (53.6)
Smoking in early pregnancy, n (%)	3 (10.7)
Multiparous, n (%)	16 (58.1)
White-Irish ethnicity, n (%)	25 (89.3)
Overweight is indicative of a BMI 25-29.9 kg/m^2<br>Obese is indicative of a BMI ≥30 kg/m^2	
